# Fitness Costs of Thermal Reaction Norms for Wing Melanisation in the Large White Butterfly (*Pieris brassicae*)

**DOI:** 10.1371/journal.pone.0090026

**Published:** 2014-02-27

**Authors:** Audrey Chaput-Bardy, Simon Ducatez, Delphine Legrand, Michel Baguette

**Affiliations:** 1 Muséum National d’Histoire Naturelle, UMR 7205 Institut Systématique Evolution Biodiversité, Paris, France; 2 INRA, Equipe Ecotoxicologie et Qualité des Milieux Aquatiques, UMR 985 Ecologie et Santé des Ecosystèmes, INRA-Agrocampus, Rennes, France; 3 Department of Biology, McGill University, Montreal, Quebec, Canada; 4 Station d’Ecologie Expérimentale du CNRS à Moulis, CNRS USR 2936, Moulis, France; Oxford Brookes University, United Kingdom

## Abstract

The large white butterfly, *Pieris brassicae*, shows a seasonal polyphenism of wing melanisation, spring individuals being darker than summer individuals. This phenotypic plasticity is supposed to be an adaptive response for thermoregulation in natural populations. However, the variation in individuals’ response, the cause of this variation (genetic, non genetic but inheritable or environmental) and its relationship with fitness remain poorly known. We tested the relationships between thermal reaction norm of wing melanisation and adult lifespan as well as female fecundity. Butterflies were reared in cold (18°C), moderate (22°C), and hot (26°C) temperatures over three generations to investigate variation in adult pigmentation and the effects of maternal thermal environment on offspring reaction norms. We found a low heritability in wing melanisation (*h^2^* = 0.18). Rearing families had contrasted thermal reaction norms. Adult lifespan of males and females from highly plastic families was shorter in individuals exposed to hot developmental temperature. Also, females from plastic families exhibited lower fecundity. We did not find any effect of maternal or grand-maternal developmental temperature on fitness. This study provides new evidence on the influence of phenotypic plasticity on life history-traits’ evolution, a crucial issue in the context of global change.

## Introduction

Knowing the mechanisms allowing organisms to cope with rapid variations of their environmental conditions is a research priority in the current era of anthropogenic global changes and species extinction [1], [2]. Adaptation is the only alternative to extinction for those organisms that are unable to move fast enough to track the shift of their climatic envelopes [3]. Adaptation occurs through two main molecular mechanisms, polymorphism of gene expression (phenotypic plasticity) and polymorphism of gene sequences (allelic fixation) [4]. Phenotypic plasticity consists in changes in phenotypic expression of a genotype in response to environmental factors [5], [6] and has been shown to have significant evolutionary consequences [7], [8]. Plasticity is adaptive if the phenotypes produced in two different environments result in higher average fitness across both environments than either fixed phenotype would [9], [10]. Adaptive phenotypic plasticity thus allows organisms to maintain fitness in rapidly changing environments [11]–[15].

In spite of this benefit, phenotypic plasticity is not ubiquitous at all [16]. Plasticity of organisms is limited in the range of environments they can respond to [17]. There are indeed some constraints such as limits or costs of plasticity that are not straightforward to show [18]. Plasticity costs are defined as any fitness reduction incurred by a plastic individual compared with a non-plastic individual that expresses the same trait value [19]. Studies attempting to quantify these costs have found either no or limited support to fitness decrease in plastic organisms [16], [18]. After a thorough review of studies that measure the costs of plasticity, Auld et al. [16] concluded that a potentially common correlation between environment-specific trait values and the magnitude of trait plasticities (i.e. multi-collinearity) could result in imprecise and/or biased estimates of costs. However, plasticity costs are expected to be common because developmental responses to environmental changes show evidence of imperfect adaptation [12]–[18]. Phenotypic plasticity creates resource allocation trade-offs during development, i.e. increased investment into one trait may decrease investments in others [17]. Given the occurrence of phenotypic plasticity and the impact that a cost of plasticity could have on phenotypic evolution, identifying costs of plasticity is thus an important issue to evolutionary ecologists [18], [20].

Phenotypic plasticity is commonly estimated as the response of an organism to an environmental gradient. Importantly, this response can be affected by environmental conditions experienced by the parents of this organism [12], [21]. Integrating sources of non genetic inheritability such as the parental environmental effects on phenotypic variation [22], [23] might thus bring information of major interest to better understand the evolution of phenotypic plasticity. Especially, we could expect non genetic transgenerational effects to affect the way organisms cope with phenotypic plasticity costs [24].

Here we tackle these issues by investigating the cost of plasticity in wing melanisation in response to temperature variation. We use the large white butterfly, *Pieris brassicae* L., as model species. This butterfly shows seasonal polyphenism, the spring morph being usually darker than the summer one because of larger melanised areas on the fore and the hind wings [25]. Studies in other Pierid butterflies have reported that wing melanisation have a high to moderate heritability [26], [27]. This variation in wing melanisation heritability may be due to genotype-environment interactions and then we can expect that those differences in mean wing melanisation can be adaptive. The synthesis of melanin, which is a complex nitrogen-rich polymer with a heavy molecular weight, involves costs in butterflies, especially since the allocation of nitrogen-rich pigment material is constrained within the closed metabolic system of a developing pupa, as shown both by various indirect and direct evidences [28]–[32]. Melanin and components of the melanin synthesis pathway ensure a wide range of functions in insects, from thermoregulation to immune defence and tegument coloration but also in traits as diverse as wound healing, cuticle sclerotisation (hardening) and egg tanning [29], [33]–[35]. Given the complexity of the melanin synthesis pathway and the various roles played by melanin in insect homeostasis, we expect significant trade-offs between the melanin production per se, and fitness-related traits [29].

In *Pieris brassicae*, the experimental exposition of full-sibling caterpillars to contrasted temperatures during their development is supposed to induce the expression of the spring or the summer morphs [25]. Such plasticity of wing melanisation in response to photoperiod and/or temperature is supposed to be adaptive in Pierid butterflies with respect to thermoregulation [26], [29], [36]. Increased melanisation may increase the direct absorption of solar radiation, thus favoring a faster heating rate or giving the ability to reach higher body temperatures. The large white butterfly typically basks by opening its wings at angles of 5–75° to the incident sunlight. This behavior allows dorsal wings to act as mirrors that reflect the solar radiation onto the thorax and/or the more melanised part of the wings [35], [37], [38]. When butterflies use reflectance basking, melanisation of the central and distal parts of the dorsal forewing is thus expected to influence body temperature [35], [37], [38]. Decreased melanisation of spots and distal parts of forewings is expected to favor the efficiency of reflectance basking [35], [37], [38]. In a related species to *P. brassicae*, *Pieris rapae*, Stoehr and Goux found a plastic response of the distal part of dorsal forewings to temperature variation [36]. In addition, dorsal forewing patterns are expected to play an important role in sexual selection and mate choice in *Pieris* butterflies. Especially, UV reflectance is known to affect mate detection and choice in *P. rapae* [39]. As UV reflectance is strongly affected by both wing melanin patterns and external light conditions [40], we may also expect variation in melanisation across time and space according to the expected light conditions [40]. Overall, dorsal wing melanisation in *P. brassicae* may thus be affected by various, potentially opposed, selection pressures. Consequently, plasticity in wing melanisation may depend on combined effects of temperature and light, and can be affected by selection on thermoregulation efficiency and sexual selection.

In this study, we address the following questions:

1. What is the relative importance of the additive genetic variation as opposed to the environmental variation in the wing melanisation? We used ‘animal model’ methods to estimate the heritability of wing melanisation and to partition the relative roles of these two sources of variation.
2. Does the level of plasticity in wing melanisation vary among full-sib families? Phenotypic plasticity was quantified from variance between environments and from the slope of the reaction norm, which is the phenotypic trait value expressed as a function of environmental conditions [13].
3. Is there a fitness cost associated with the plasticity in wing melanisation (after taking into account direct costs associated with mean melanisation), and is it affected by non-genetic parental effects? We assumed plasticity costs would appear when the strength of the reaction norm negatively affects fitness independently from the character values expressed within single environments [19].

## Materials and Methods

### Ethics Statement

No permits were necessary for the sampling of individuals and the experimental work on the large white butterfly, and this project did not involve endangered or protected species.

### Rearing Experiment

Experiments were performed at the Muséum National d’Histoire Naturelle in Brunoy, France, using butterflies from a recently established breeding. The stock originated from eggs collected in Brunoy (Essonne, France, 48°42′00″N, 2°30′00″E), Moulis (Ariège, France, 42°57′40″N, 01°05′27″E) and Mesnil-Eglise (Belgium, 50°10′00″N, 45°58′00″E) during the summer 2007.

Twenty mating pairs were isolated from the tenth generation of captive breeding, 1507 eggs were collected from these mating pairs and served as basis for the first generation of our experiment (F1). The rearing experiment consisted of three temperature treatments over three generations to estimate melanin heritability and to test the existence of transgenerational effects of parental and grand-parental developmental temperature on offspring plasticity. Full-sibling eggs were randomly divided over three climate rooms set to constant temperatures of 18°C (cold treatment), 22°C (moderate treatment), and 26°C (hot treatment). Optimum temperature for development seems to be between 20°C and 26°C [41]. Moreover, a pilot experiment with F9 individuals showed that larvae reared above 26°C and below 18°C experienced high mortality (>90%). Larvae were separated in familial groups of thirty individuals. They were reared on cabbage, *Brassica oleracea* provided *ad libitum*, in 15*9*9 cm boxes under a constant light cycle (Light : Dark 14∶ 10 h) that induces direct development without pupal diapause. Temperature, light brightness and hygrometry were controlled daily within each climate rooms (POL-EKO ST500), using the same thermometer, hygrometer and light meter, and the only significant difference we observed between the rooms was the temperature (which is the manipulated variable here). Rearing boxes with caterpillars were moved from their position in a climate room every day to avoid a position effect in the room. After emergence, imagoes were placed in 60*60*60 breeding cages, at 25°C ±1°C with unrelated individuals, where their survival was recorded daily. Mating pairs were isolated in 20*20*20 cm laying cages to produce the next generation (F2). Every day, the eggs laid by each female on cabbage leaves were counted before splitting them over the three temperature treatments. Eggs from the same clutches were full siblings as females could mate only once. The number of offspring at each developmental stage was also recorded. We used the same protocol to produce the next generation (F3). The developmental temperature of F1 and F2 generations was manipulated in order to test the importance of maternal and grand-maternal effects on F3 generation, while the developmental temperature of F3 generation was manipulated to test potential costs of phenotypic plasticity.

### Picture Acquisition and Processing

We developed a system to take standardised digital photography of living imagoes after their emergence. Each individual was photographed on the day of its emergence in order to limit wing attrition effect on colouration and area of wing. Butterflies were anaesthetized with nitrogen monoxide using an Inject+Matic Sleeper TAS and held between two transparent plastic pieces. The butterfly was then placed into a light tent on a standard white background. The tent diffused homogeneous and constant light from two lamps on both sides. Grey reflectance standards were included in pictures at the start of a photography session [42]. A decimeter arranged under the butterfly allowed us to control the scale of each photograph. The digital camera was placed on a tripod and in a dark room at a distance of 30 cm from the butterfly. We used a Nikon D300 digital camera equipped with a 105 mm macro lens. The shutter lag time was 10 seconds to impede vibrations. We manually set the white balance according to the light intensity and we maintained a constant integration time and a constant lens aperture [42].

The left and the right forewings were extracted from the digital picture (4288×2488 pixels) with the Gimp shareware (http://gimp.org/), and wing variables were measured with the ImageJ shareware (http://rsb.info.nih.gov/ij/). The total areas of the right and left forewing of each individual were measured. Melanised areas had a grey reflectance score above the threshold of 120 (where 0 = white, 255 = black). This threshold reliably separated melanised from unmelanised scales on the wings according to the grey reflectance standards [36]. Based on a subsample of 30 individuals, wing measures were highly repeatable (99% of the variation in repeated measurements, including repeated photographing, is due to between individual variations).

### Inheritance of Wing Melanisation

**Figure 1:**
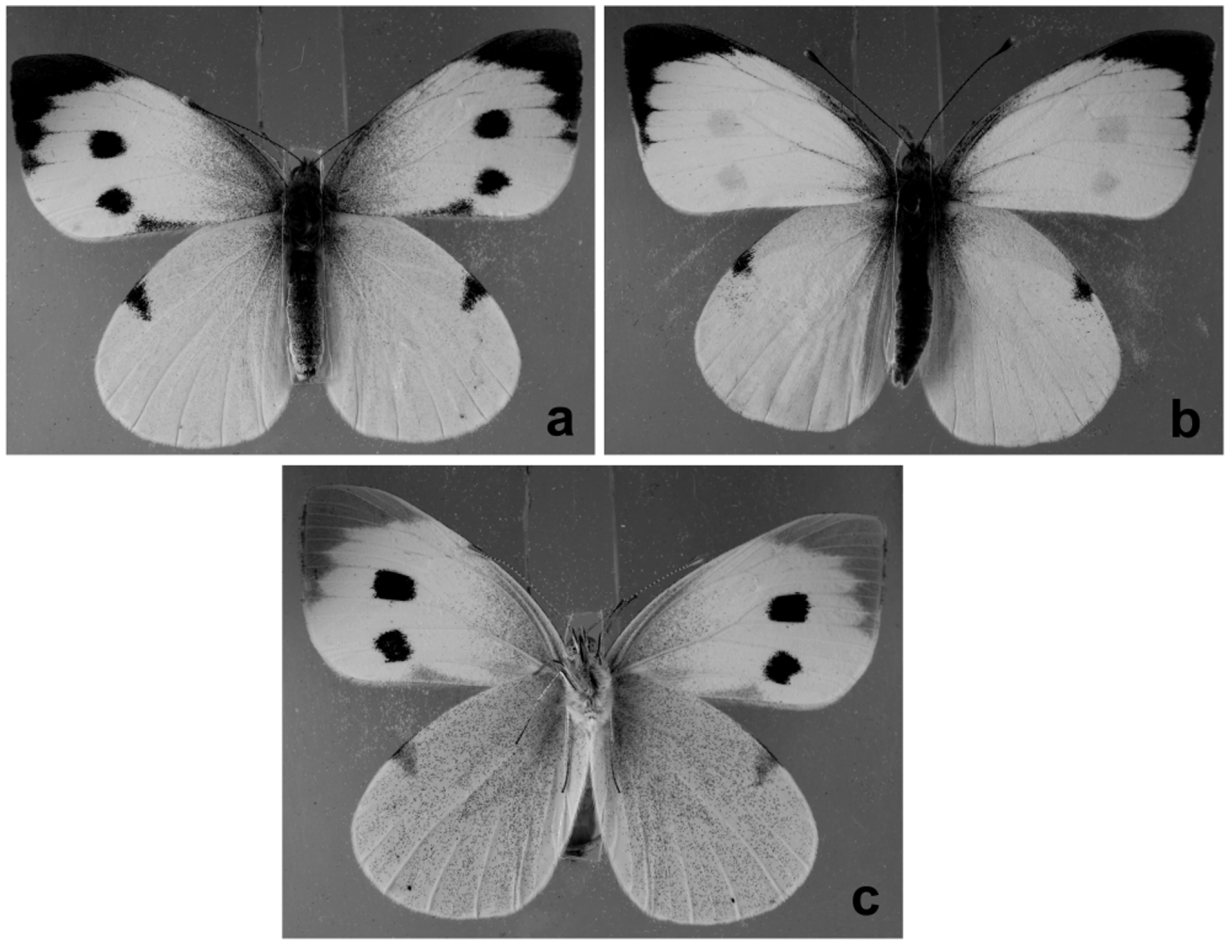
Dorsal wings of female (a) and male (b) *Pieris brassicae*. The colouration of ventral wings is the same for both sexes; a female is shown here (c). Total black areas of dorsal and ventral forewings were measured.

A total of 829 butterflies (403 males and 426 females) on 3 000 harvested eggs over three generations were photographed. Adult size was estimated by averaging the area of left and right forewings (i.e. ‘size’ variable). Total black area was also averaged over both forewings for each side (i.e. ventral and dorsal, see Figure 1). As ventral melanised area was correlated with dorsal melanised area (Pearson’s correlation test, r^2^ = 0.87, t = 51.21, df = 827, p<0.0001), we used dorsal melanised area for subsequent analyses.

We estimated wing melanisation heritability from the pedigree data (829 butterflies over 3 generations) fitting animal models with the R package MCMCglmm [43]. The animal model is a mixed-effect model that allows assessing the genetic and non genetic components of phenotypic variation. Individuals’ pedigree is included as an explanatory random variable to estimate genetic variance. This approach allowed to partition the additive genetic variance (animal effect), the variance due to maternal effects, and the residual environmental variance of melanisation. The forewing melanised area constituted the response variable. Forewing area and sex were treated as fixed effects and animal effect (pedigree), rearing temperature, mother temperature and grand-mother temperature were treated as random effects. The MCMC chains were run for 1 300 000 iterations with a burn-in period of 300 000 to ensure satisfactory convergence [44]. Parameters, estimated standard errors and confidence intervals were performed by sampling 1 000 times the posterior parameter distribution. The deviance information criterion (DIC) was used for model selection. The difference between DIC-values of two competing models should not exceed 10 [45]. Adding a random effect ‘rearing box’ nested in the animal effect did not improve the models, and we thus excluded this effect from the models.

### Wing Melanisation Plasticity

For analyses of wing melanisation plasticity we used the offspring of 9 females (9 families, 585 individuals) from the F3 generation. Thereby we measured wing melanisation plasticity at the family level. As estimators of phenotypic plasticity, we considered: (i) the variance of melanin proportion (i.e. the ratio between dorsal melanised area and total wing area) between temperatures, and (ii) the absolute slope of the linear regression between the melanin proportion and temperature in each family. We used the absolute slope of the reaction norm, as we were interested in the magnitude of the variation, and the direction of the variation was indicated by a supplementary binary variable (i.e. the ‘slope sign’).

We tested the effect of wing melanisation plasticity for dorsal forewings on adult lifespan, using linear mixed effect models (LME) with sex, melanin proportion, rearing temperature, rearing cage, rearing temperature of the mother and the grand-mother, mating status and first order interactions as supplementary fixed effects, and family as a random effect. The mating status, hereafter called ‘mate’ indicates whether an individual mated or not. This variable is known to negatively affect adult lifespan due to an energetic cost [46], [47]. The melanin proportion was added to models in order to verify that lifespan was influenced by melanin plasticity and not by individual melanisation only. We performed a model selection approach using the Akaike Information Criterion (AIC, [48]) and a model averaging procedure. Akaike weights (*Wi*) that represent the relative probability for a model *i* to be the best among considered models, were calculated for the subset of models having (AICbest −AICi) ≤2. In a model averaging procedure, averaged parameters and their corresponding unconditional standard errors were calculated from the smallest subset of AIC-ranked models for which ∑*Wi* was ≥0.95. The relative importance of each variable within the averaged model was estimated by adding *Wi-values* of those models within the 95% confidence set containing that variable [48]–[50].

We tested the effect of wing melanisation plasticity on female fecundity (30 females from F3 generation) using generalized linear mixed models (GLMM) with the number of hatching eggs as response variable, and wing melanisation plasticity, melanin proportion, rearing temperature, rearing temperatures of the mother and the grand-mother, female lifespan and their interactions as fixed effects, and family as a random effect. The number of hatching eggs was analyzed using a quasi-Poisson error and a logarithmic function link (a Poisson GLM correcting for overdispersion [51]). As in quasi-Poisson models, the AIC is not defined, we performed analysis of deviance to compare two nested models (full and nested models [52]). The best model was obtained by stepwise deletion of non significant terms (p>0.05). No transformations of response variables were needed to meet the assumptions of normality and homoscedasticity. Statistical analyses were performed using R, version 2.11.1 (The R Foundation for Statistical Computing, Vienna).

## Results

### Heritability of Wing Melanisation

The most parsimonious model included forewing area and sex as fixed effects and animal effect and rearing temperature as random effects (Table 1). The animal model provided evidence for equivalent effects of additive genetic variation (CVa = 40.03; 95% Highest Posterior Density Interval (HDPI) = 20.00–62.26), developmental temperature variation (CV = 35.19; 95% HDPI = 10.13–527.08) and environmental (residual) variation (CVe = 48.91; 95% HDPI = 36.54–60.98) on wing melanisation, which resulted in a rather low heritability estimate for melanin (*h^2^* = 0.18; HDPI = 0.01–0.41). It is noteworthy that maternal and grand-maternal temperatures were not included in the best model.

**Table 1:** Model selection for ‘animal models’ of melanin variation to estimate forewing melanisation heritability.

Random effect	Fixed effect	DIC
–	–	7444.30
animal	–	7413.91
animal+RT	–	7381.75
animal+RT+MT	–	7389.82
animal +RT+MT+GMT	–	7402.16
animal+RT	FA	7030.22
animal+RT	sex	6352.47
**animal+RT**	**FA+sex**	**5759.95**

Model selection is based on deviance information criterion (DIC). Forewing melanisation heritability was estimated from the most parsimonious model in bold.

**RT** is the rearing temperature.

**MT** is the mother rearing temperature.

**GMT** is the grand-mother rearing temperature.

**FA** is the forewing area.

### Thermal Reaction Norm

**Figure 2:**
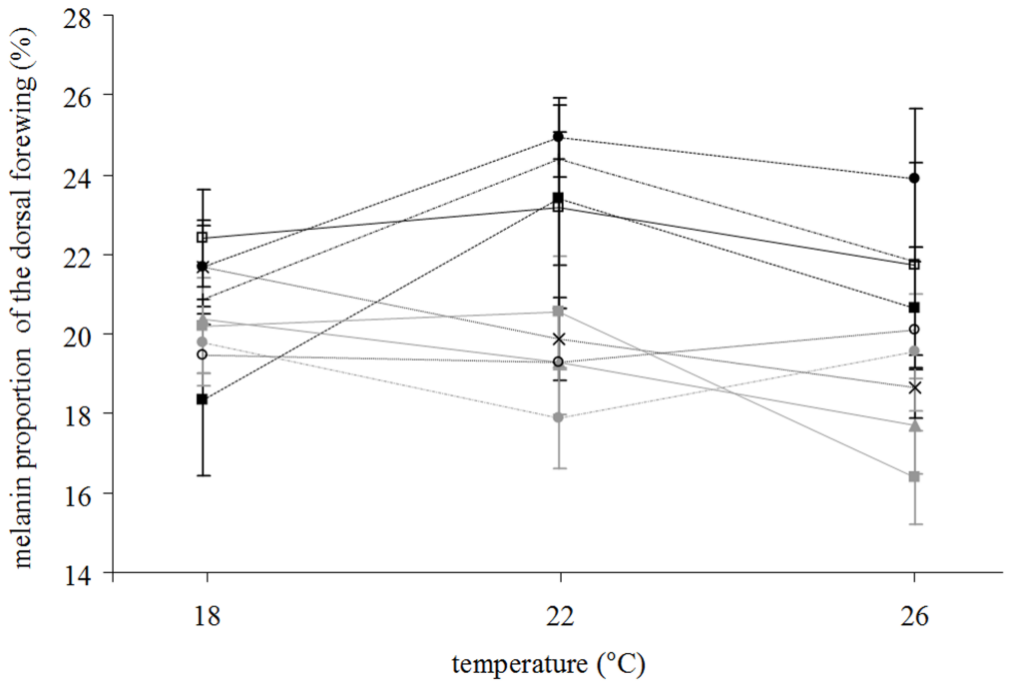
Thermal reaction norms (mean ± standard error) for melanin proportion of the dorsal forewing in 9 full-sib families of *Pieris brassicae*.

The 9 families showed contrasted reaction norms (i.e. the relationship between melanin and temperature, Figure 2). Mean reaction norms exhibited positive, flat and negative slopes (Figure 2). Testing the effect of interaction between temperature and family on wing melanisation indicated that variations between environments were significantly different across families (Likelihood Ratio Chi-Square = 1190, DF = 8, p<0.0001). Wing melanisation increased significantly with temperature in 4 families (0.006< slope of the reaction norm <0.021), decreased significantly in 4 families (−0.007< slope of the reaction norm<−0.027), and did not significantly vary in one family (slope = −0.002, p = 0.136, Table 2).

**Table 2:** Effect of rearing temperature on melanin proportion in 9 butterfly families (GLM results).

*Family 1*	Estimate	SE	z	p
Intercept	−1.624	0.0202	−80.2	<0.0001
Temperature	0.021	0.0009	22.19	<0.0001
*Family 2*				
Intercept	−1.7402	0.0299	−58.22	<0.0001
Temperature	0.0184	0.0013	13.67	<0.0001
*Family 3*				
Intercept	−0.9731	0.0251	−38.82	<0.0001
Temperature	−0.0214	0.0012	−18.35	<0.0001
*Family 4*				
Intercept	−0.8607	0.0184	−46.66	<0.0001
Temperature	−0.0236	0.0008	−28.55	<0.0001
*Family 5*				
Intercept	−0.8563	0.0222	−38.5	<0.0001
Temperature	−0.0274	0.0011	−25.84	<0.0001
*Family 6*	Estimate	SE	z	p
Intercept	−1.5451	0.0156	−99.35	<0.0001
Temperature	0.0062	0.0007	9.36	<0.0001
*Family 7*				
Intercept	−1.2953	0.0231	−56.07	<0.0001
Temperature	−0.0069	0.0011	−6.24	<0.0001
*Family 8*				
Intercept	−1.2003	0.0274	−43.8	<0.0001
Temperature	−0.0021	0.0014	−1.49	0.136
*Family 9*				
Intercept	−1.5584	0.023	−67.88	<0.0001
Temperature	0.0128	0.0012	10.57	<0.0001

‘Estimate’ corresponds to the slope of the thermal reaction norm and SE stands for the standard error.

### Effect of Plasticity on Adult Lifespan

We did not find a parental or grand-parental effect of rearing temperature on adult lifespan, as mother and grand-mother rearing temperatures were not included as random factors in the best models. Wing melanisation plasticity (the slope of the reaction norm and the variance between environments), melanin proportion, rearing temperature, sex, mating status and interactions accounted for adult lifespan (Table 3). Only the magnitude of the thermal reaction norm (the absolute slope of the reaction norm) accounted for wing melanisation plasticity, as the direction of the reaction norm (the slope sign) did not affect lifespan.

**Table 3:** Effects of forewing melanisation plasticity (‘plasticity’), melanin proportion (‘melanin’), sex, mating and rearing temperature on lifespan in *Pieris brassicae*: results of model averaging on linear mixed models with family as a random effect.

(a) plasticity = variance between environments						
Parameter	Estimate	SE	Adjusted SE	z	p	Relative importance
Intercept	2.92E+01	1.96E+01	1.96E+01	1.491	0.1361	1
mate	−1.09E+01	9.32E+00	9.33E+00	1.168	0.2428	1
melanin	−1.03E+00	7.47E-01	7.49E-01	1.373	0.1699	0.83
**sex**	**−1.97E+01**	**8.81E+00**	**8.83E+00**	**2.233**	**0.0255**	**1**
**plasticity**	**9.47E-06**	**4.01E-06**	**4.60E-06**	**2.058**	**0.0396**	**1**
rearing temperature	−6.40E-01	9.42E-01	9.44E-01	0.678	0.4976	1
**mate:sex**	**6.95E+00**	**3.59E+00**	**3.60E+00**	**1.934**	**0.0531**	**1**
mate:plasticity	4.17E-06	2.48E-06	2.48E-06	1.677	0.0935	0.72
melanin:temperature	6.05E-02	3.24E-02	3.24E-02	1.865	0.0622	0.77
**sex:plasticity**	**2.05E-06**	**9.85E-07**	**9.87E-07**	**2.075**	**0.0379**	**0.73**
sex:temperature	7.90E-01	4.07E-01	4.07E-01	1.938	0.05257	1
**plasticity:temperature**	**−4.83E-07**	**1.48E-07**	**1.48E-07**	**3.258**	**0.0011**	**1**
melanin:plasticity	−1.65E-07	8.37E-08	8.38E-08	1.969	0.0489	0.27
melanin:sex	−2.54E-01	2.46E-01	2.46E-01	1.033	0.3015	0.16
mate:melanin	5.08E-01	4.99E-01	5.00E-01	1.016	0.3096	0.19
mate:temperature	3.52E-01	3.55E-01	3.56E-01	0.989	0.3228	0.19
**(b) plasticity = slope of the reaction norm**						
**Parameter**	**Estimate**	**SE**	**Adjusted SE**	**z**	**p**	**Relative importance**
Intercept	7.66806	15.34079	15.35699	0.499	0.6175	
mate	−14.59819	13.81057	13.82346	1.056	0.2909	1
melanin	−0.07471	0.63756	0.63822	0.117	0.9068	0.81
sex	−12.59627	8.20778	8.21625	1.533	0.1252	1
plasticity	720.96612	356.4199	403.42805	1.787	0.0739	1
rearing temperature	0.41018	0.69892	0.6996	0.586	0.5577	1
mate:sex	7.87137	4.42812	4.43427	1.775	0.0759	0.88
mate:plasticity	268.41227	154.4337	154.73693	1.735	0.0828	0.65
melanin:temperature	0.02767	0.03843	0.03847	0.719	0.4719	0.35
**sex:plasticity**	**191.65618**	**99.01677**	**99.20913**	**1.932**	**0.0534**	**0.7**
sex:temperature	0.50927	0.31558	0.31601	1.612	0.1071	0.8
**plasticity:temperature**	**−34.87847**	**10.44949**	**10.47155**	**3.331**	**0.0009**	**1**
melanin:plasticity	−14.21438	10.79973	10.8175	1.314	0.1888	0.43
melanin:sex	−0.19636	0.24174	0.24225	0.811	0.4176	0.26
mate:melanin	0.45264	0.59386	0.59464	0.761	0.4465	0.36
mate:temperature	0.3734	0.39313	0.39384	0.948	0.3431	0.37

Plasticity is estimated by the variance of forewing melanisation between temperatures (a) and the absolute slope of the thermal reaction norm (b). Females were taken as references for the calculation of coefficients. Significant variables (p<0.05) with a relative importance >0.60 are bolded. SE stands for the standard error.

**Figure 3:**
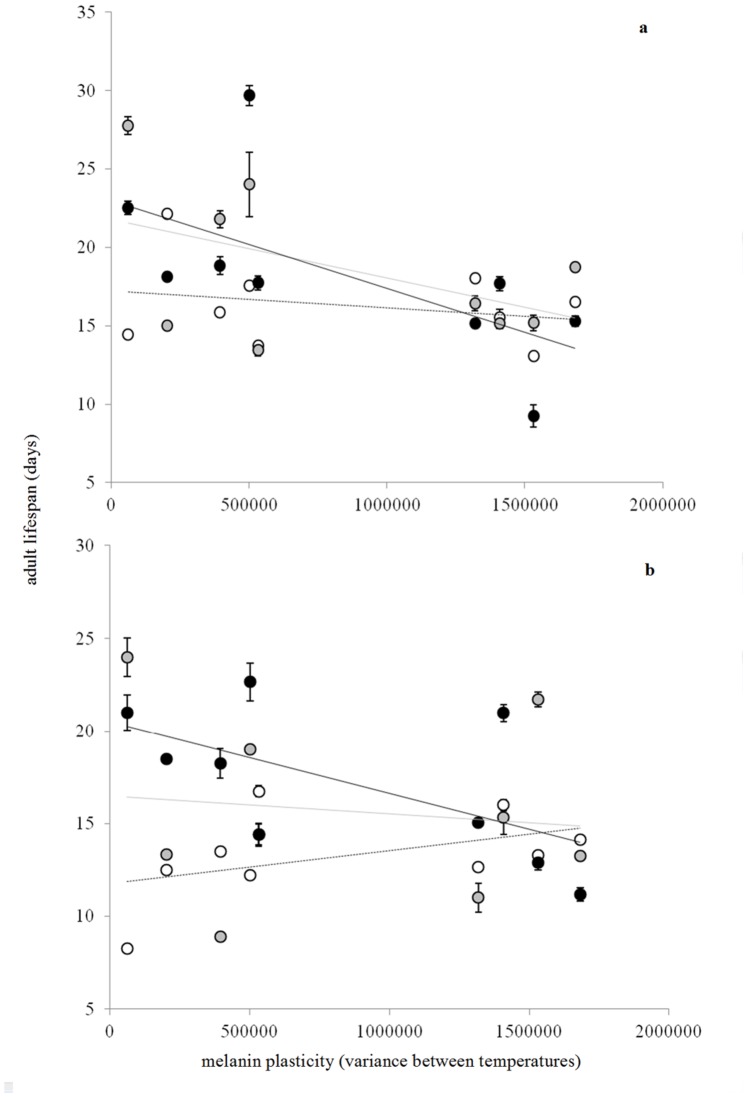
Effect of the interaction between wing melanisation plasticity (i.e. variance between environments) and rearing temperature on mean adult lifespan (± standard error) in females (a) and males (b). The continuous black line and closed circles correspond to 26°C, the continuous grey line and grey circles to 22°C and the dotted black line and open circles to 18°C.

In order to better understand the effects of interactions between melanin plasticity, sex and rearing temperature on lifespan (Table 3), we made post-hoc analyses by sex within each temperature. These post-hoc analyses were linear models with lifespan as response variable and plasticity (i.e. variance between environments) as fixed effect (Table 4 and Figure 3). Lifespan was differently affected by temperature according to the sex. The lifespan of males tended to be longer in high wing melanisation plasticity families at 18°C and shorter at 26°C (Table 4 and Figure 3). The lifespan of females was also shorter at 26°C when plasticity increased (Table 4 and Figure 3).

**Table 4:** Effect of forewing melanisation plasticity (i.e. within family variance in forewings melanisation between temperatures) on lifespan in females and males at a rearing temperature of 18, 22 and 26°C.

Sex	Rearing temperature	Estimate	SE	t	p
females	18	−1.30E-06	1.03E-06	−1.261	0.2090
	22	−1.54E-07	1.36E-06	−0.113	0.9100
	26	−3.05E-06	1.24E-06	−2.457	0.0159
males	18	1.74E-06	9.32E-07	1.864	0.0647
	22	1.65E-06	1.57E-06	1.048	0.2990
	26	−3.58E-06	1.37E-06	−2.621	0.0104

SE stands for the standard error.

### Effect of Plasticity on Females’ Fecundity

Females’ fecundity was also negatively influenced by wing melanisation plasticity although this effect was weaker considering variance of melanin between temperatures instead of the slope of the thermal reaction norm (Table 5). There was no developmental and parental effect of temperature on fecundity. Females from plastic families produced fewer eggs. Females that lived longer produced more eggs than those with a shorter lifespan (Table 5).

**Table 5:** Effects of forewing melanisation plasticity and female lifespan on fecundity.

(a) plasticity = variance between environments				
Parameter	Estimate	SE	t	p
Intercept	4.42E+00	4.24E-01	10.433	<0.0001
lifespan	5.53E-02	2.46E-02	2.249	0.0329
plasticity	−9.29E-07	4.93E-07	−1.884	0.0704
**(b) plasticity = slope of the reaction norm**				
**Parameter**	**Estimate**	**SE**	**t**	**p**
Intercept	4.65115	0.43775	10.625	<0.0001
lifespan	0.05701	0.02422	2.353	0.0261
plasticity	−65.86106	27.25042	−2.417	0.0227

Here is presented the most parsimonious model. Plasticity is estimated by the variance of forewing melanisation between temperatures (a) and the absolute slope of the thermal reaction norm (b). We used a GLMM and applied a deviance analysis for model selection (see Methods). SE stands for the standard error.

## Discussion

Roff [53] compared the heritability of suites of life history, behavioural, physiological and morphological traits in a large panel of organisms and showed that life-history traits are always less heritable than other traits. We report here a rather low heritability of wing melanisation in *P. brassicae* (*h* ^2^ = 0.18), which is in the range of heritabilities of life-history traits rather than of morphological or physiological traits. This result is in contradiction with studies on other Pierids that showed a high to moderate heritability of melanin [26], [27]. We suggest that this low heritability of melanin deposition on the wings reflects more developmental plasticity associated with the multiple functions of melanin in the homeostasis of insects [29], [33]–[35] rather than the transmission of pure morphological or physiological attributes. The effect of environment on wing melanisation revealed here by the animal model is also in line with adaptive plasticity reported in Pierid butterflies with respect to thermoregulation [26], [29], [36]. This adaptive plasticity does not seem to be influenced by the developmental temperature experienced by the previous generations. This result confirms findings of other cross-generation studies in which effects of parental temperature on offspring life-history traits were weak or absent [54], [55].

Wing melanisation is classically supposed to decrease with increasing temperature [56], [57]. Here we found that some butterfly families were darker at warmer temperatures. Besides, the reaction norms of these 9 families were markedly different. Our results seem to support a quantitative genetic variation in thermal reaction norms for wing melanisation, rather than a simple, general mechanistic relationship linking temperature to melanin deposition. Families of a same population can diverge not only in the average amount of plasticity expressed but also in their patterns of inter-individual variation in plasticity. The maintenance of this variation may be explained by the existence of different life history and ecological strategies within populations, or by differences in individuals’ exposure to selective pressures in the wild. Such a reversal in the temperature-phenotype rule, together with between-family variation in thermal reaction norms were also observed for the body size in natural populations of *Pieris rapae* [58]. Adaptive plasticity consists in producing an optimum phenotype corresponding to the prevalent local environmental conditions [59]. We would expect individuals that experienced particular environmental conditions during their larval development to be better adapted to similar conditions during their adult stage. However, we showed here that adult lifespan was not higher when larvae developed at 26°C, which is close to adult rearing temperature (25°C ±1°C). Similarly to ours, studies on thermal reaction norms based on the breeding of larvae at contrasted temperatures [29], [36], [58] investigated the adaptability of adult phenotypes at only one temperature regime. Alongside with the recommendation of Gilchrist and Huey [55], we suggest that it would be highly informative to study the relationship between life-history traits and temperature on adults manipulating adult breeding temperatures according to the ranges of developmental temperatures.

We have highlighted the coexistence of high and low wing melanisation plasticity families in our experimental population (Figure 2). Among males, high plasticity seemed to confer a lifespan benefit at low temperature but not at moderate temperature, and became costly at high temperature. Among females, high wing melanisation plasticity incurred a lifespan cost at high temperature and a fecundity cost regardless the developmental temperature. Overall, in our experimental setting, wing melanisation plasticity was costly for females, whereas males seemed to benefit from plasticity at low temperature. The evolutionary dynamics of this potential trade-off certainly deserves further research; we suggest again that this exciting issue could be fully addressed only by comparing fitness of adults bred under the same ranges of temperatures than those experienced by larvae during their growth.

Here, adult butterflies experienced a homogeneous thermal environment across 13 generations in rearing conditions. Such stable conditions probably maintained constant the costs of wing melanisation plasticity. Differences in fitness between high and low wing melanisation plasticity families tend to confirm that homogeneous environmental conditions could select against plasticity or favour canalisation [18]. Plastic individuals must indeed invest resources in maintaining the molecular/physiological ‘machinery’ needed to detect, monitor and respond to various environmental conditions [16] and therefore may be counter-selected under stable conditions.

Temperature is one of the environmental cues that influences the expression of many phenotypic traits [13]. We reported here on fitness costs of thermal reaction norms for wing melanisation in a butterfly, which potentially entail selection against the more plastic individuals. Making quantitative predictions on the extent to which the homogenisation of the environmental conditions [60] associated with the current era of global changes will affect phenotypic plasticity is thus an important challenge.
